# Outcomes with bridging radiation therapy prior to chimeric antigen receptor T-cell therapy in patients with aggressive large B-cell lymphomas

**DOI:** 10.3389/fimmu.2025.1517348

**Published:** 2025-01-31

**Authors:** Gohar S. Manzar, Chelsea C. Pinnix, Stephanie O. Dudzinski, Kathryn E. Marqueen, Elaine E. Cha, Lewis F. Nasr, Alison K. Yoder, Michael K. Rooney, Paolo Strati, Sairah Ahmed, Chijioke Nze, Ranjit Nair, Luis E. Fayad, Michael Wang, Loretta J. Nastoupil, Jason R. Westin, Christopher R. Flowers, Sattva S. Neelapu, Jillian R. Gunther, Bouthaina S. Dabaja, Susan Y. Wu, Penny Q. Fang

**Affiliations:** ^1^ Department of Radiation Oncology, The University of Texas MD Anderson Cancer Center, Houston, TX, United States; ^2^ Department of Lymphoma/Myeloma, The University of Texas MD Anderson Cancer Center, Houston, TX, United States

**Keywords:** CAR-T, chimeric antigen receptor, radiation therapy, bridging RT, DLBCL

## Abstract

**Background:**

Select patients with relapsed/refractory aggressive B cell lymphoma may benefit from bridging radiation (bRT) prior to anti-CD19-directed chimeric antigen receptor T cell therapy (CAR-T). Here, we examined patient and treatment factors associated with outcomes and patterns of failure after bRT and CAR-T.

**Methods:**

We retrospectively reviewed adults with diffuse large B-cell lymphoma (DLBCL) who received bRT prior to axicabtagene ciloleucel, tisagenlecleucel, or lisocabtagene maraleucel between 11/2017-4/2023. Clinical/treatment characteristics, response, and toxicity were extracted. Survival was modeled using Kaplan-Meier or Cox regression models for events distributed over time, or binary logistic regression for disease response. Fisher’s Exact Test or Mann-Whitney U methods were used.

**Results:**

Of 51 patients, 25.5% had bulky disease and 64.7% had Stage III/IV disease at the time of RT. Comprehensive bRT alone to all disease sites was delivered to 51% of patients, and 29.4% were additionally bridged with systemic therapy. Median follow-up was 10.3 months (95% CI: 7.7-16.4). Overall response rate (ORR) was 82.4% at 30 days post-CAR-T infusion. Median overall survival (OS) was 22.1 months (6.6-not reached) and the median progression-free survival (PFS) was 7.4 months (5.5-30). OS/PFS were 80% (66-99)/78% (64-87) at 1-year, and 59% (44-71)/54% (40-67) at 2-years, respectively. Comprehensive RT to all sites of disease correlated with improved PFS and OS, *p ≤* 0.04. Additionally, ECOG ≥2 and Stage III/IV disease predicted poor OS (*p ≤* 0.02). Disease bulk, IPI ≥3, and non-GCB histology were poor predictors for disease-specific survival (DSS), *p*<0.05. The latter two, as well as bRT dose of ≤30 Gy predicted worse PFS (*p*<0.05). Among patients with advanced stage disease, comprehensive bRT to all sites of disease (*n*=10) was not associated with improved OS and PFS compared to focal bRT (*n*=23), *p*>0.17. No difference was seen in bridging RT vs. chemoRT. Twenty-six patients developed relapse (50.9%), of which 46% was in-field. Risk of in-field relapse correlated with bulky disease (OR=7, 95% CI: 1.2-41, *p*=0.03) and lack of response at 30 day post-CAR-T evaluation (OR=16.8, 95% CI: 1.6-176, *p*=0.02), but not with bRT dose (*p*=0.27).

**Conclusion:**

bRT and CART is a good treatment strategy for select patients with aggressive B cell lymphoma. Comprehensive bRT including all sites of disease is associated with improved outcomes.

## 1 Introduction

Chimeric antigen receptor (CAR) T-cell therapy targeting CD19 has revolutionized the treatment of relapsed or refractory B cell lymphomas. It is being adopted earlier in treatment paradigms considering recent favorable outcomes in second-line trials ZUMA-7 and TRANSFORM (1–4). Three autologous CD19-directed CAR T-cell therapies (tisagenlecleucel, axicabtagene ciloleucel, and lisocabtagene maraleucel) are currently approved for the treatment of diffuse-large B cell lymphoma (DLBCL) on the basis of encouraging initial overall response rates (ORRs), with lasting responses achieved in 40-74% of patients across landmark clinical trials. However, up to 60% of patients do not attain a CR after undergoing CAR T-cell therapy, and up to 70% of patients eventually experience progressive disease (PD) with very poor outcomes (5, 6). In turn, there is a compelling motivation to identify treatment approaches that may improve CAR-T cell efficacy.

Many patients planned to undergo CAR T-cell therapy harbor symptomatic, active disease that could be fatally progressive if left unmanaged during the 3- to 4-week autologous cell-manufacturing phase. For these patients, “bridging” therapy delivered between leukapheresis and CAR-T infusion can be beneficial. Bridging therapy can be also given to patients due to perceived high risk of relapse, palliation, or for cytoreduction. The array of options for bridging therapy encompasses radiotherapy (RT), chemotherapy, steroids, and immunotherapy, potentially in various combinations. There is limited data available to guide the selection among these modalities, with resulting heterogeneity and wide variance in clinical practice.

Bridging RT (bRT) as an approach is an appealing modality due to its efficacy in cases of chemorefractory disease, the known sensitivity of hematological malignancies to RT, and effective disease debulking, as well as potential for synergy and immune priming by radiation (7). With the advent of additional cellular therapies and earlier incorporation of CAR T-cell therapy in relapsed/refractory treatment paradigms due to favorable clinical trial results with CAR T-cell therapy, there is increased applicability of optimizing bridging strategies, including bRT, as this approach will likely become more relevant.

There is a paucity of data to guide the selection, treatment, and timing of bRT prior to CAR-T therapy. Prior data demonstrates that bRT is safe and does not impose additional toxicity (8), does not compromise the therapeutic efficacy of CAR T-cell therapy, and may improve outcomes such as progression-free survival (9), potentially by converting patients with high-risk features associated with post-CAR-T failure into a better risk category (10). However, what has been published so far includes single-institution series with practice variations and small numbers of patients, most ranging from n=3-17 (8, 9, 11, 12), with a few larger series (n=41 or n=375) (10, 13). Several unanswered or unverified questions remain that would benefit from cross-institutional validation. These questions include which patients benefit most from bRT, ideal bRT regimens, and optimal design of treatment fields, particularly for patients with multifocal disease.

Herein, we present our institutional experience of 51 patients with DLBCL who underwent bRT prior to CD19-directed CAR T-cell therapy, analyzing patterns of failure and examining the patient and treatment factors associated with the most benefit from bRT.

## 2 Materials and methods

### 2.1 Study population

With IRB approval, we retrospectively reviewed patients age ≥18 with pathologically confirmed DLBCL treated at a single institution between 11/2017-4/2023 who received bRT prior to commercial CAR-T cell therapy, which included axicabtagene ciloleucel, tisagenlecleucel, or lisocabtagene maraleucel. Clinicopathologic features, treatment characteristics, response, and toxicity were extracted.

### 2.2 Bridging radiation treatment

Patients typically underwent multidisciplinary evaluation with a recommendation for bridging RT (defined as the period between leukapheresis up until the time of lymphodepleting chemotherapy (cyclophosphamide [500 mg/m2] and fludarabine [30 mg/m2] administered on days −5, −4, and −3). If bRT commenced prior to leukapheresis but continued during the period between T-cell collection and the infusion of CAR-T cell therapy, it was also categorized as bRT. bRT was sometimes given with concurrent systemic therapy, which included chemotherapy, steroids, or targeted agents. RT was photon-based with IMRT/VMAT or 2D/3D conformal radiation at the discretion of the treating physician. bRT fields were outlined on a case-by-case basis, but typically involved-site, and categorized as “comprehensive” if all sites of disease were treated, or otherwise “focal” if not.

### 2.3 Data collection

Medical records were reviewed and the following demographic, disease, and treatment information were extracted: age at time of bRT, sex, ECOG at leukapheresis, histopathologic data, comorbidities, Ann Arbor disease stage at the time of apheresis, International Prognostic Index (IPI) score, bulky disease (defined as nodal/extranodal conglomerate measuring ≥7.5 cm in maximum dimension), number and nature of prior lines, site of disease, lab features at leukapheresis, radiation treatment details, dates of leukapheresis, CAR-T infusion, and bridging therapy, symptoms at the time of RT. Hospitalization details and adverse events were recorded prospectively, with toxicity grading for the severity of cytokine release syndrome (CRS) and immune effector cell–associated neurotoxicity syndrome (ICANS) according to consensus CARTOX criteria until 4/2019 and thereafter according to criteria issued by the American Society for Transplantation and Cellular Therapy. Evaluation of disease response was generally performed 30 days post-CAR-T infusion using positron emission tomography-computed tomography (PET-CT) incorporating Lugano classification. For further outcome assessment, we recorded response at 90 days, 6 months, 9 months, and 12 months, with relevant Deauville five-point response scores. In cases of relapse or disease progression, we noted any clinical or radiographic findings, and identified the site of failure in relation to the radiation fields. Finally, we documented the disease status and vitality of the patients at their last follow-up, including the cause of death, where applicable.

### 2.4 Statistical analysis

Demographic characteristics were examined with descriptive statistics using Mann-Whitney U for continuous variables and Chi-square tests for categorical variables. Overall survival (OS) was calculated by determining the duration from CAR-T infusion to death resulting from any cause. Progression-free survival (PFS) was estimated by computing the duration from CAR-T infusion until objective tumor progression or relapse by imaging or biopsy, or death of any cause. Disease-specific survival (DSS) was calculated by determining the duration from CAR-T infusion to death resulting from lymphoma. Kaplan-Meier curves visualized survival trends, and the evaluation of survival disparities was conducted using a log-rank test. Patient status was marked as “censored” upon reaching the date of the latest follow-up or arriving at 5/1/2023, depending on whichever event came first. Univariate analyses were generated for age (<60 vs. ≥60), tumor bulk (<7.5 cm vs. ≥7.5 cm), IPI score (1-2 vs. 3-5), ECOG performance status (0-1 vs. ≥2), double or triple expressor, double or triple hit, GCB or non-GCB, ≥3 prior lines of therapy, disease stage (I-II vs. III-IV, also written as limited vs. advanced) at the time of apheresis, “localized” disease able to be encompassed in RT fields (even if Stage IV) vs. “extensive” disease not able to be incorporated fully by RT, type of bridging (RT vs. chemoRT), receipt of RT focally vs. comprehensively to all sites of disease, bRT starting either pre- or post-leukapheresis, and bRT dose (<30 vs. ≥30 Gy). We also compared outcomes following focal vs. comprehensive RT in patients with advanced stage disease at apheresis, and in patients with bulky tumors, for whom we also investigated RT dose. Statistical analyses and graphs were generated using Prism v9.0 (GraphPad, La Jolla, CA) or SPSS Statistics (IBM, Armonk, NY, USA). The swimmer’s plot was generated in R v4.3.1 (Vienna, Austria). All comparisons were 2-sided.

## 3 Results

### 3.1 Patient and tumor characteristics

Fifty-one patients received bRT (**Table 1**). The median age was 65, with 60.1% of patients ≥60 years of age. Most patients were male. Thirteen patients (25.5%) had bulky disease (≥7.5 cm) at the time of bRT. The cohort primarily consisted of patients with an IPI score of 2 or 3 (*n*=33). Patients received a median of 2 prior therapies (range: 1-4), including 5 with autologous stem cell transplant and 2 with immune checkpoint inhibitors (ICI). Over half of the patients (51%, *n*=25) received three or more prior lines of therapy. Eighteen (35.3%) patients had limited stage disease at apheresis, compared to 33 patients with Stage III/IV disease (64.7%). Accounting for disease location from a radiotherapeutic feasibility standpoint, 27 patients (52.9%) had localized disease (even if Stage IV by official staging) and 24 (47.1%) had extensive disease.

**Table 1:** Patient demographics and treatment characteristics at the time of leukapheresis.

Patient characteristics	*n*	%
Total No.	51	
Age		
Median (range), years	65 (24 — 87)
Sex		
Male	36	70.6%
Female	15	29.4%
ECOG		
0	5	9.8%
1	33	64.7%
2	10	19.6%
3	3	5.9%
4	0	0.0%
Disease type		
DLBCL	40	78.4%
PMBCL	4	7.8%
Transformed FL	7	13.7%
Stage		
Limited (I/II)	18	35.3%
Advanced (III/IV)	33	64.7%
Site(s) of disease		
Localized	27	52.9%
Extensive	24	47.1%
IPI score		
1	6	11.8%
2	14	27.5%
3	19	37.3%
4	7	13.7%
5	5	9.8%
IPI score category		
≤2	21	41.2%
≥3	30	58.8%
High-risk features		
Bulky disease	13	25.5%
≥3 prior therapies	25	51.0%
Primary refractory	29	56.9%
History of CNS disease	8	15.7%
Double or triple expressor / high-grade B cell lymphoma with Myc and Bcl translocations*		
Expressor via IHC		
Yes	20	39.2%
No	28	54.9%
Unknown	3	5.9%
Translocations via FISH		
Yes	11	21.6%
No	39	76.5%
Unknown	1	19.6%
Subtype		
GCB	37	72.5%
Non-GCB	9	17.6%
Unknown	5	9.8%
Bridging treatment type		
RT alone	35	68.6%
Chemoradiation	16	31.4%
CAR-T product		
Axicabtagene ciloleucel (Yescarta)	40	78.4%
Lisocabtagene maraleucel (Breyanzi)	9	17.7%
Tisagenlecleucel (Kymriah)	2	3.9%
Radiation to all sites of disease?		
Yes, comprehensive bRT	26	51.0%
No, focal RT	25	49.0%
Leukapheresis timing relative to bRT		
Before bRT (post-leukapheresis bRT)	43	84.3%
After bRT (pre-leukapheresis bRT)	8	15.7%
Radiation treatment		
Median dose (range), Gy	30 (4 — 48)
Fractions (range)	10 (2 — 23)
Other treatments		
Prior auto-SCT	5	9.8%
Therapy lines prior to CAR-T median (range)	2 (1 — 4)	

*Double-hit = MYC^+^/BCL-2^+^; Triple-hit = MYC^+^/BCL-2^+^/BCL-6^+^.

Expressor refers to protein overexpression without translocations.

### 3.2 Bridging RT details

All three commercially available and approved CAR-T cell products were utilized in this study, but axicabtagene ciloleucel was the most common (*n*=40, 78.4%). Most patients (*n*=43, 84.3%) received bRT that started post-leukapheresis, with median (IQR) duration between the last day of RT and CAR infusion of 18 days (12.5-25). bRT was delivered with a median dose of 30 Gy (range: 4-48 Gy). bRT regimens were heterogenous (**Supplementary Table 1**), but the most common was 20 Gy in 8 fractions (*n*=7), as well as 30 Gy in either 12 fractions (*n*=4) or 10 fractions (*n*=3), with a median EQD2 of 30.09 (range: 4-52). Twenty-six patients (51%) received ≥30 Gy. Majority received bRT alone (*n*=31, 68.6%), and 16 (31.4%) were additionally bridged with systemic therapy. A list of these systemic therapies is shown in **Supplementary Table 2**.

Twenty-six patients (51%) received bRT comprehensively to all active disease sites. The majority of patients with limited stage disease were treated comprehensively with bRT (*n*=16, 88.9% of limited stage patients) compared to less than half of the patients with ≥Stage III disease (*n*=10, 30.3% of the advanced stage patients), *p*<0.001. Among patients with localized disease, 74.1% received comprehensive bRT (*n*=25 of 27), compared to only 4.2% of patients with extensive disease (*n*=1 of 24), *p*<0.001. Those who received bRT comprehensively received a median (IQR) dose of 30 Gy (20.6-40.9) in 10 (8-17) fractions, compared to 23.4 Gy (18-37.5) in 10 (6-15) fractions, *p*=0.25.

### 3.3 Overview of clinical outcomes

**Figure 1:**
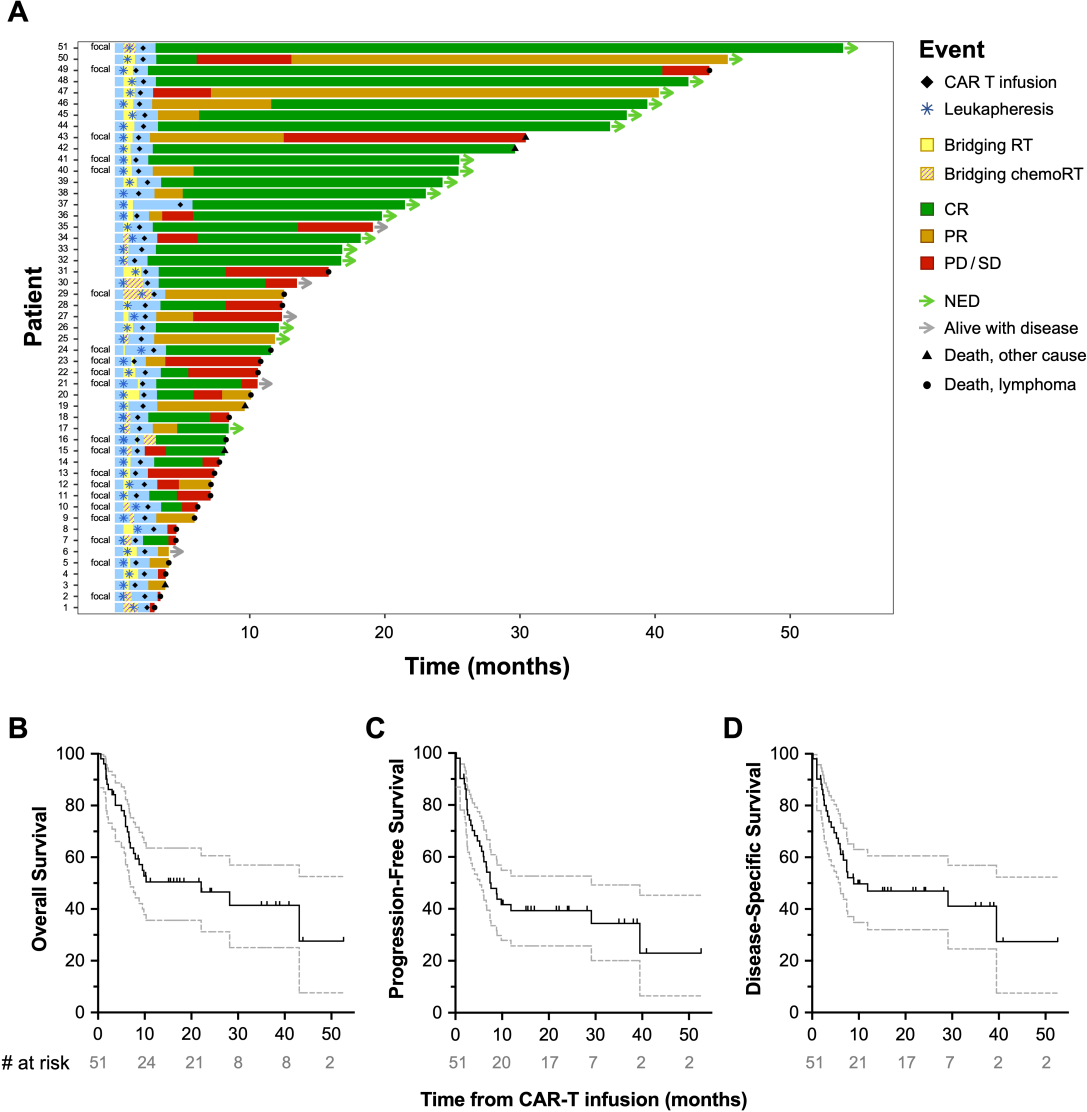
Patient outcomes post-CAR-T infusion. Swimmer’s plot depicting the course of individual patients following CAR-T infusion **(A)** for the full study cohort (*n*=51). The median OS was 10.3 months **(B)**, median PFS was 7.4 months **(C)**, and median DSS was 8.9 months **(D)**.

Regarding outcomes, the objective response rate (ORR) was 82.4% at 30 days post-CAR-T infusion. This comprised of 26 patients (51%) with complete response (CR) and 16 (31.4%) with partial response (PR), of whom 7 patients eventually developed CR—4 at three months, 2 at six months, and 1 at nine months (**Figure 1A**). Nine patients (17.6%) had progression of disease (PD) or stable disease (SD). Of 26 patients who experienced CR, 13 (50%) eventually relapsed—5 at three months, 6 at six months, and 2 at twelve months. Twenty-seven patients (52.9%) remain alive at last follow-up, 19 (70.4%) of whom have no evidence of disease (NED). The median OS (**Figure 1B**) was 22.1 months (6.6-not reached), median PFS (**Figure 1C**) was 7.4 months (5.5-30), and median DSS (**Figure 1D**) was 8.9 months (6-not reached). OS, PFS, and DSS were 80% (66-99), 78% (64-87), and 82% (68-90) at 1-year, and 59% (44-71), 54% (40-67), and 59% (44-71) at 2-years, respectively. After CART, 4 patients (7.8%) experienced Grade ≥3 cytokine release syndrome (CRS), 17 (33.3%) had Grade ≥2 CRS, and 21 (41.2%) had Grade ≥3 neurotoxicity. No severe adverse events in the RT field were noted.

### 3.4 Patterns of first failure following CAR T-cell therapy in patients bridged with RT

**Figure 2:**
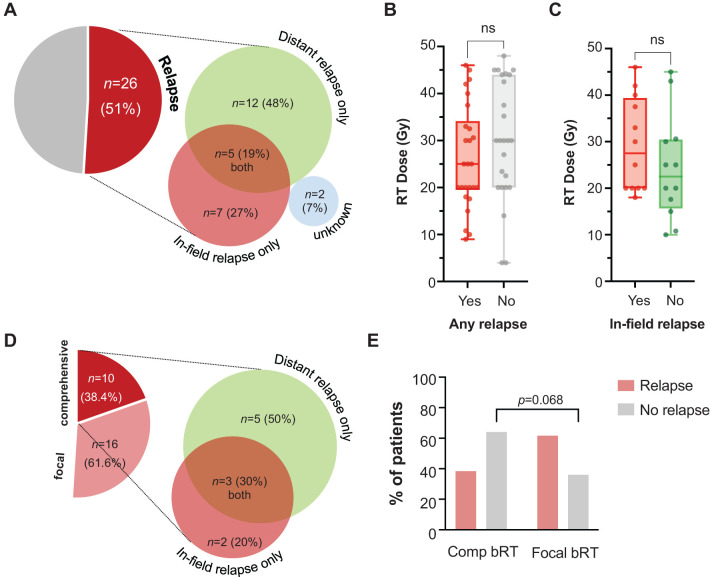
Incidence and nature of relapse and relationship with dose or extent of RT. Relapse was evident in n=26 patients (51%), with type of relapse shown in proportionally-accurate diagrams **(A)**. RT dose is plotted for individual patients, categorized by whether they experienced any relapse **(B)**. Only among patients who developed relapse, RT dose was plotted against emergence of in-field vs. distant relapse **(C)**. Among patients who relapsed following receipt of comprehensive bRT to all sites of disease, the category of relapse is depicted **(D)**. The incidence of relapse in patients who received comprehensive or focal bRT is also shown **(E)**.

At a median follow-up of 10.3 months (95% CI: 7.7-16.4) from leukapheresis, 26 patients developed relapse (50.9%), **Figure 2A**. Of these patients, 12 (48%) had only distant relapses, 7 (27%) in-field only, 5 (19%) had both in- and out-of-field relapses, and 2 (7.7%) unknown. Dose <30 Gy (both nominal dose and EQD2) did not associate with increased likelihood of in-field relapse, *p*>0.27. Dose ≥30 Gy was given to 60% of patients without any relapse (*n*=15/25), compared to 50% of patients with in-field relapses (*n*=6/12). Median dose (IQR) was 25 Gy (20-33) for patients with relapse, and 30 Gy (20-44) for those without relapse (**Figure 2B**). Patients with in-field relapse received a median (IQR) of 27.5 Gy (20-38.2) compared with 22.5 Gy (17-30.2) for those who developed out-of-field only relapse (**Figure 2C**). Among the 26 patients who relapsed, only 38.4% (*n*=10) were treated with comprehensive bRT. Of these 10 patients, 5 (50%) developed out-of-field relapse, 2 (20%) in-field only relapse, and 3 patients (30%) developed both in- and out-of-field relapses (**Figure 2D**). This was a similar proportion to the larger cohort of relapses. In contrast, among patients who did not develop relapse (*n*=25), 64% (*n*=16) were treated comprehensively with bRT to all sites of disease, *p*=0.068 (**Figure 2E**). Among the 16 patients with limited stage disease treated comprehensively, 11 did not relapse (68.75%). Among the 5 patients who relapsed, 3 (50%) developed out-of-field relapse, and 2 patients (33.3%) developed both in- and out-of-field relapses (treated to 20 Gy in 8 fractions and 42 Gy in 21 fractions).

On univariate analysis, in-field relapse was associated with bulky disease (OR=7, [95% CI: 1.2-41], *p*=0.03) and lack of response at the 30-day post-CAR-T evaluation (OR=16.8, [95% CI: 1.6-176], *p*=0.02). It did not correlate with IPI score (*p*=0.97), double/triple expressor or high-grade B cell lymphoma with Myc and Bcl2 translocations (*p*=0.94), disease stage (*p*=0.97), localized vs. extensive disease (*p*=0.41), receipt of systemic therapy with bRT (*p*=0.5), bRT timing pre- or post-leukapheresis (*p*=0.9), bRT dose of ≥30 Gy (*p*=0.28), or receipt of comprehensive or focal bRT (*p*>0.9).

### 3.5 Univariate analysis of factors associated with worse OS, PFS, and DSS

**Figure 3:**
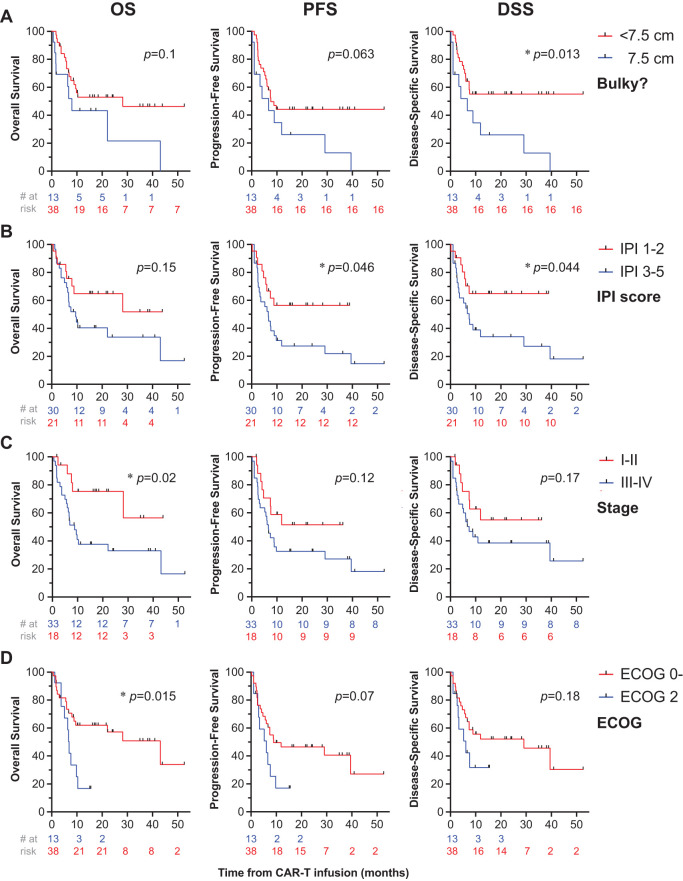
Univariate analysis of disease- and patient-related factors associated with survival. OS (first column), PFS (second column), and DSS (third column) were computed for patients who got bridging RT or chemoradiation prior to CAR-T cell therapy. Patients were stratified by patient or disease-related factors including tumor bulk **(A)**, IPI score **(B)**, disease stage **(C)**, and ECOG status **(D)**, with * denoting *p*<0.05.

**Figure 4:**
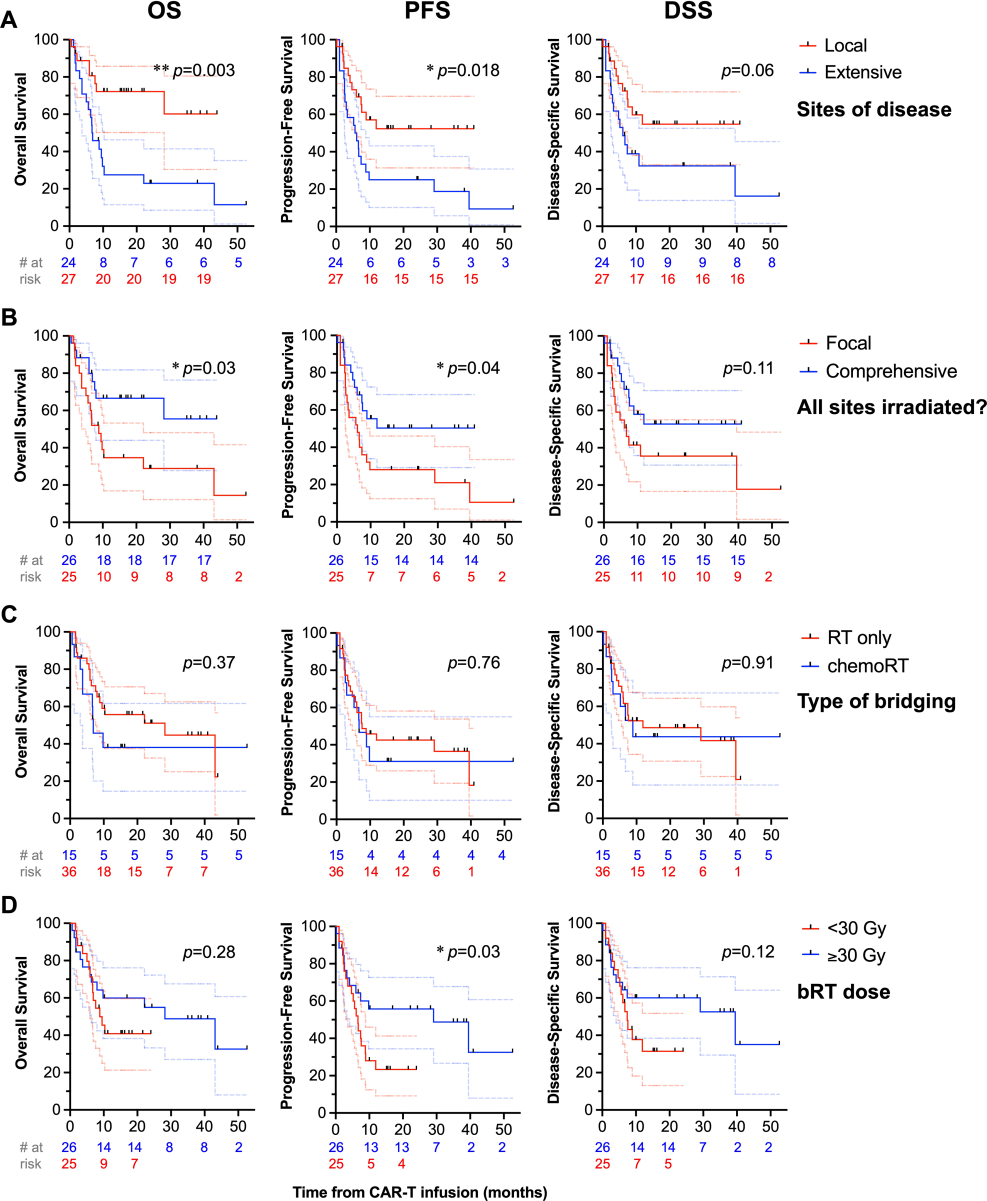
Univariate analysis of treatment-related factors associated with survival. OS (first column), PFS (second column), and DSS (third column) are depicted with treatment-related factors, such as disease category as localized vs. extensive **(A)**, receipt of bRT comprehensively to all sites of disease or to focal areas of disease **(B)**, bridging strategy **(C)**, and bRT dose **(D)**, with * denoting *p*<0.05.

On univariate analysis, disease bulk (≥7.5 cm) was associated with reduced DSS (1-year DSS: 13% [1-41] vs. 73% [55-84], *p*=0.01) and trended toward worse PFS (1-year PFS: 13% [1-41] vs. 68% [51-81], *p*=0.06), but not OS (*p*=0.1), **Figure 3A**. IPI ≥3 was associated with worse PFS (1-year PFS: 56% [33-75] vs. 62% [42-77], *p*=0.046) and worse DSS (*p*=0.044), but not OS (*p*=0.15), **Figure 3B**. Non-GCB histology correlated with worse PFS (1-year PFS: 11% [1-39] vs. 73% [57-84], *p*=0.025) and DSS (1-year DSS: 15% [1-47] vs. 78% [61-88], *p*=0.03), but not OS (*p*=0.1). Factors associated with significant decrements in OS included Stage III/IV disease (1-year OS: 38% [21-54] vs. 75% [47-90], *p*=0.02), **Figure 3C**, ECOG ≥2 (1-year OS: 17% [3-41] vs. 71% [53-83], p=0.015), **Figure 3D**, and if disease was categorized as being diffuse as opposed to localized (1-year OS: 23% [8-42] vs. 72% [50-86], *p*=0.003), **Figure 4A**. Stage III/IV disease did not significantly associate with reduced PFS (1-year: 33 [17-49] vs. 52 [26-72], *p*=0.12) or DSS (1-year: 39 [21-56] vs. 55 [27-76], *p*=0.17), **Figure 3C**. Extensive spread of disease did associate with worse PFS (1-year: 25 [10-43] vs. 52 [31-70], *p*=0.018) but only a trend toward reduced DSS (1-year: 32 [14-52] vs. 55 [33-72], *p*=0.06), **Figure 4A**.

**Figure 5:**
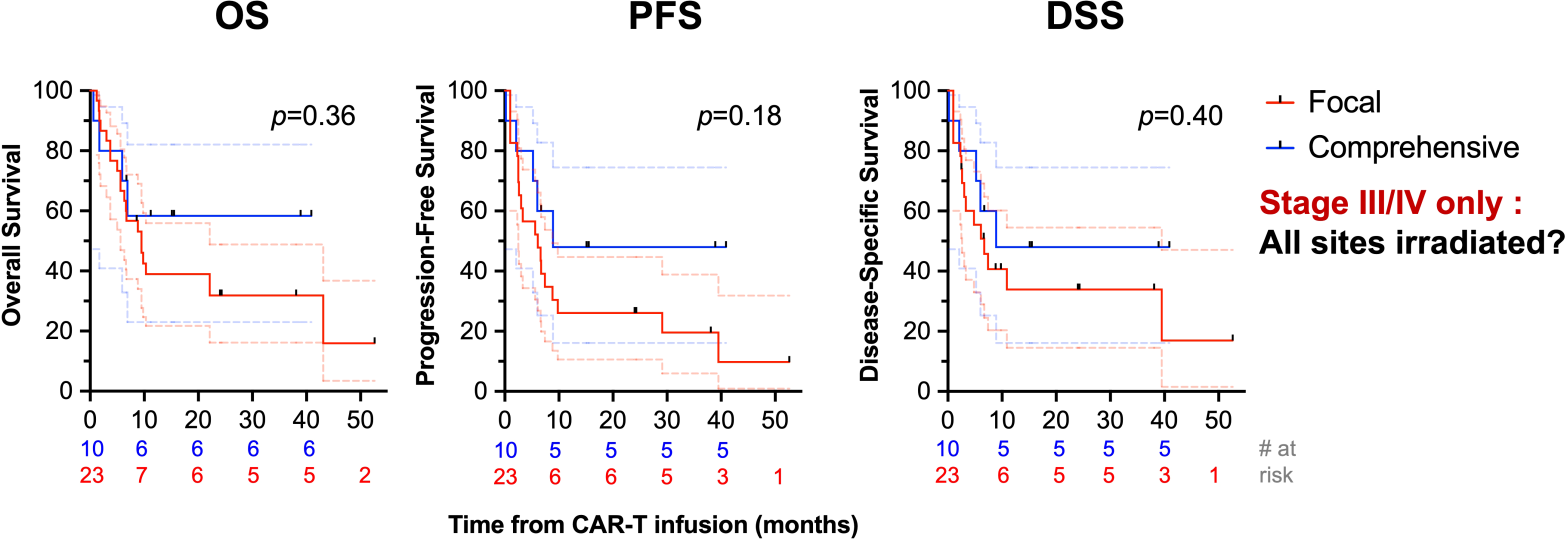
Univariate analysis of survival stratified by comprehensiveness of bRT fields in patients with advanced stage disease. OS (first column), PFS (second column), and DSS (third column) are depicted comparing receipt of bRT comprehensively to all sites of disease or to focal areas of disease in patients with advanced stage disease.

Receipt of RT comprehensively to all sites of disease correlated with improved PFS (1-year PFS: 50% [29-68] vs. 28% [12-46], *p*=0.04) and increased OS (1-year OS: 67% [44-82] vs. 34% [16-53], *p*=0.03), **Figure 4B**. Among patients with bulky tumors (*n*=13), there was no association of focal vs. comprehensive bRT fields (**Supplementary Figure 1**) or bRT dose of ≥30 Gy (**Supplementary Figure 2**) with differences in OS, PFS, or DSS (*p*>0.2). There was no difference in PFS, OS, or DSS between patients who received bRT or chemoRT (*p*>0.3), **Figure 4C**. Receipt of ≥30 Gy bRT correlated with improved PFS (2-year: 33% [8-61] vs. 18% [6-36], *p*=0.03) but not OS (*p*>0.25) or DSS (*p*>0.1), **Figure 4D**. Additionally, no differences in OS, PFS, or DSS were noted with stratification by age ≥60 (*p*>0.15), double or triple expressor or high grade B cell lymphoma with Bcl-2 and Myc translocations (*p*>0.51), ≥3 prior lines of therapy (*p*>0.23), or receipt of bRT pre- or post-leukapheresis (*p*>0.67). There was not an OS (*p*=0.18), PFS (*p*=0.18), or DSS (*p*=0.4) benefit in patients with advanced stage disease (*n*=33) treated with comprehensive bRT (*n*=10) vs. focal bRT (*n*=23), **Figure 5**. Of 27 patients with localized disease, only 2 received focal bRT, and of 24 patients with extensive disease, only 1 patient received comprehensive bRT, which did not make comparison of outcomes with comprehensive vs. focal bRT in the localized or extensive disease subsets feasible.

Of 18 patients with limited stage disease, 16 were treated with comprehensive bRT and two patients were treated with focal bRT. One patient had relapsed primary mediastinal B cell lymphoma (PMBCL) with dyspnea from the mediastinal mass, and she was referred for bridging chemoRT with a planned prescription of 30 Gy in 15 fractions. A small right subpectoral node was not included in the treatment field encompassing the mediastinal mass. She ultimately completed 14 Gy in 7 fractions before RT was held for one week prior to leukapheresis and then CAR-T infusion approximately 1 month later. She had a CR at her 3 month post-CAR-T scan and continues to maintain NED at her most recent follow-up. The other patient had DLBCL involving the oropharynx, causing dysphagia, as well as bilateral neck nodes with greater burden on the left side. He underwent bRT alone to the oropharynx and left neck only to 25 Gy in 10 fractions; the right cervical lymph nodes were not treated. He had a partial response at his 30 day post-CAR-T scan, with continued progression of disease at 3 months in the oropharynx and neck confirmed via biopsy. He was enrolled on a clinical trial of a humanized monoclonal bispecific antibody (Plamotamab), and at most recent follow-up, continues to be alive with disease.

## 4 Discussion

We demonstrate that bRT as a precursor to CD19-directed CAR T-cell therapy can be an effective strategy for disease control in patients with relapsed/refractory aggressive B cell lymphoma. We find that comprehensive bRT is associated with improved OS and PFS but this may be due to its association with limited disease stage in patients receiving comprehensive bRT. When examining patterns of disease relapse, we found that in-field relapse was associated with bulky disease, validating prior findings (12). While bridging RT dose did not correlate with in-field relapse, we noted that receipt of ≥30 Gy bRT correlated with improved PFS. Following this, it appears that definitive doses should be favorably considered in bRT regimens, but further confirmatory, prospective studies with larger cohorts would be helpful to further elucidate these findings.

Our study adds to the limited body of work suggesting that bRT for CAR-T therapy is not only safe but also has the potential to enhance treatment outcomes in specific patient populations. The ORR of our cohort was 82.4% at 30 days post-CAR-T infusion with a 51% initial CR rate, similar to the 81.8% ORR at 30 days and better than the 27% initial CR rate found in a pilot retrospective series of 11 patients who received bRT prior to CAR T-cell therapy (11). The median OS was not reached, with 1-year OS of 80% and a 2-year OS of 59%. Qualitatively, these rates compare favorably to the 1-year OS of 58% observed in the TRANSCEND trial (3), the 2-year OS of 50.5% in the ZUMA-1 study (2), prospective studies without bRT, and it aligns closely with the 1-year survival rate of 67-80% documented in earlier, smaller bRT retrospective cohorts (8, 10, 12, 14). As expected, factors correlating with decreased OS were higher ECOG and advanced stage disease. DSS was worse with increased tumor bulk, elevated IPI, and non-GCB histology. As noted above, decrements in PFS correlated with <30 Gy bRT, as well as elevated IPI and non-GCB histology. The median PFS of 7.4 months and 1-year PFS of 78% reported in this series aligns with the median PFS described in TRANSCEND and ZUMA-1, ranging from 5.9-6.8 months (2, 3).

The predominant pattern of failure post-CAR-T has been described to involve progression or relapse at pre-existing sites of disease in both bRT and no-bridging therapy groups (12). Regarding treatment dose, we found that while receipt of ≥30 Gy bRT correlated with improved PFS, bridging RT dose did not correlate with in-field relapse. Ladbury et al. described in a recent report that no local treatment failures were seen among patients treated with bRT to a dose of > 32.5 Gy, and improved local control observed in patients receiving an EQD2 of ≥20 Gy (15). While the optimal dose of bRT remains unknown, we believe it is likely that patients with limited stage disease, when bridged with comprehensive RT, may stand to benefit the most from more definitive RT treatment doses ≥30 Gy. Prospective studies examining larger series of patients will be instrumental to validate these findings.

Comprehensive RT has been previously associated with clinical benefit in the post-CAR-T setting. In a prior report, salvage RT following CAR T-cell failure was associated with improved freedom from subsequent progression (FFSP), freedom from subsequent event (FFSE), and OS for patients who received this salvage RT in a “comprehensive” manner encompassing all active disease, as opposed to “focal” RT targeting only a limited portion of a patient’s disease (14). Initially, groups reported trends demonstrating the potential superiority of “comprehensive” RT when delivered in the bridging period but did not reach statistically significance, possibly due to the small cohorts of patients analyzed (9, 10). Within a group of 12 patients who received bridging RT, one group demonstrated statistically significant benefit in PFS and OS with comprehensive bRT compared to focal bRT (16). Compared to no bridging therapy, comprehensive bRT was demonstrated to double the rate of sustained CR without subsequent relapse, decrease local relapse, and increase event-free survival among patients with <5 pre-CAR-T sites of disease (17). In our study, patients with limited stage disease were almost exclusively treated with comprehensive bRT, preventing statistical analysis against focal bRT in this patient population. Additionally, comprehensive bRT did not improve outcomes for patients with advanced stage disease in our cohort. This contrasts a multi-institutional ILROG analysis that was presented in abstract form, demonstrating superior PFS with comprehensive bRT when controlling for disease burden—this was a multivariable model controlling for disease stage, ECOG, age, CNS disease, metabolic tumor volume (MTV), receipt of axi-cel (vs. tisa-cel), and pre-leukapheresis LDH (18).

While bRT appears to be both well-tolerated and effective, the most significant limitation of our study is the absence of a comparative control group with patients who have limited stage disease who did not receive bRT or were bridged with systemic therapy alone. Fortunately, recent data convincingly addresses this question, demonstrating enhanced disease control and durable response with comprehensive bRT (n=34) compared to no bridging therapy (n=66) among patients with less than 5 pre-CART disease sites (17). Patients who did not receive bridging therapy had a higher risk of local relapse (41% vs. 15% with bRT) as well as overall disease progression, despite harboring smaller tumors, highlighting the potential benefit of bRT in enhancing local control and altering relapse patterns (17).

We acknowledge other limitations of our study in its retrospective design. Radiation disposition, intent, dosage, and fractionation were ultimately the physician’s choice, although all cases were reviewed in a group quality assurance conference. There is selection bias inherent to this non-randomized cohort consisting of patients who likely were felt to have higher-risk or progressive lesions, for which local therapy was felt potentially beneficial pre-CAR-T infusion. Secondly, there was variability in the timing of bRT in relation to CAR T-cell infusion, and its impact on treatment efficacy is unclear. Third, the use or type of concurrent chemotherapy was not standardized. A notable fraction (31.6%) received systemic therapy with bRT, which may impact outcomes. Fourth, certain risk factors, such as MTV and LDH levels, which could predict relapse or worse prognosis, were not available or included in the Cox regression models. Finally, this cohort represents a single institution experience, potentially limiting generalizability.

Our prior reported experience involved 124 patients receiving axi-cel, with half of them undergoing bridging therapy. There was no significant difference in 1-year PFS and OS between patients eventually receiving CAR-T who underwent bridging RT and those who did not, but systemic bridging alone appeared inferior to RT with respect to 1-year PFS (8.9 with RT vs. 4.7 months with systemic therapy alone, p=0.05). In that prior report, too there was encouragingly an early signal showing a trend toward improved PFS with comprehensive bRT (p=0.12), a trend consistently supported in subsequent research and now statistically significant in our expanded cohort. Importantly, there were no discernible differences in toxicity. Our prior experience also highlighted the efficacy of RT as a bridging approach with all RT-bridged patients ultimately proceeding to CAR-T infusion. Despite the challenging prognosis often associated with the need for bridging therapy, bRT can serve as an effective strategy in this context.

To conclude, RT as a bridging approach for CAR T-cell therapy is feasible, with high post-CAR overall response rate and favorable survival identified in this cohort of heavily pre-treated patients with DLBCL. When feasible, comprehensive bRT, which we show correlates with improved efficacy, should be considered, particularly in patients with limited stage disease prior to apheresis. Future prospective research is essential to evaluate the potential benefits of bRT, to confirm the optimal bRT strategy and further investigate the potential for enhanced immune synergy between CAR-T and RT.

## Data Availability

The raw data supporting the conclusions of this article will be made available by the authors, without undue reservation.
