# Supplementary material for: Outcomes with bridging radiation therapy prior to chimeric antigen receptor T-cell therapy in patients with aggressive large B-cell lymphomas
Source: Front Immunol. 2025 Jan 31;16:1517348. doi: 10.3389/fimmu.2025.1517348 (PMC11825444; doi:10.3389/fimmu.2025.1517348)

Supplementary Material

**Supplementary Table 1.** Heterogenous bRT regimens used in this cohort.

| **Regimen (Dose / Fractions)** | **Number of patients** |
| --- | --- |
| 20 Gy / 8 | 7 (13.7%) |
| 30 Gy / 12 | 4 (7.8%) |
| 45 Gy / 18 | 4 (7.8%) |
| 25 Gy / 10 | 3 (5.9%) |
| 30 Gy / 10 | 3 (5.9%) |
| 4 Gy / 2 | 2 (3.9%) |
| 37.5 Gy / 15 | 2 (3.9%) |
| 9 Gy / 3 | 1 (1.96%) |
| 10 Gy / 5 | 1 (1.96%) |
| 10.8 Gy / 6 | 1 (1.96%) |
| 14 Gy / 7 | 1 (1.96%) |
| 15 Gy / 6 | 1 (1.96%) |
| 17.6 Gy / 8 | 1 (1.96%) |
| 18 Gy / 6 | 1 (1.96%) |
| 20 Gy / 4 | 1 (1.96%) |
| 20 Gy / 5 | 1 (1.96%) |
| 20 Gy / 10 | 1 (1.96%) |
| 22.5 Gy / 9 | 1 (1.96%) |
| 23.4 Gy / 13 | 1 (1.96%) |
| 27 Gy / 9 | 1 (1.96%) |
| 30.6 Gy / 17 | 1 (1.96%) |
| 32.5 Gy / 13 | 1 (1.96%) |
| 33 Gy / 15 | 1 (1.96%) |
| 35.2 Gy / 16 | 1 (1.96%) |
| 40 Gy / 16 | 1 (1.96%) |
| 42 Gy / 21 | 1 (1.96%) |
| 42.5 Gy / 17 | 1 (1.96%) |
| 43 Gy / 16 | 1 (1.96%) |
| 44 Gy / 20 | 1 (1.96%) |
| 44 Gy / 22 | 1 (1.96%) |
| 44.2 Gy / 17 | 1 (1.96%) |
| 46 Gy / 23 | 1 (1.96%) |
| 48 Gy / 16 | 1 (1.96%) |

**Supplementary Table 2.** List of systemic therapies used in the bridging period prior to CAR-T cell infusion.

| **No. of patients** | **Type of systemic bridging therapy** |
| --- | --- |
| 4 | Polatuzumab vedotin, rituximab |
| 1 | Ibrutinib |
| 1 | Nivolumab, brentuximab |
| 1 | Obintuzumab, cyclophosphamide, dexamethasone |
| 1 | Obintuzumab, lenalidomide, dexamethasone |
| 1 | High-dose cytarabine, daratumumab |
| 1 | Polatuzumab vedotin, Bendamustine, rituximab (Pola-BR) |
| 1 | Rituximab |
| 1 | Rituximab, cyclophosphamide |
| 1 | Rituximab, cyclophosphamide, dexamethasone |
| 1 | Rituximab, dexamethasone, cytarabine, cisplatin |
| 1 | Rituximab, gemcitabine, oxaliplatin |
| 1 | Rituximab, paclitaxel, topotecan |
| **16** | **TOTAL** |

**Supplementary Figure 1. Comparing survival in patients specifically with bulky disease who received focal vs. comprehensive bRT.** Kaplan-Meier curves illustrating the OS **(A)**, PFS **(B)**, and DSS **(C)** among only patients with bulky tumors (*n*=13) treated comprehensively (*n*=8) vs. focally (*n*=5) with bridging RT.

**Supplementary Figure 2. Comparing survival in patients specifically with bulky disease who received <30 Gy vs. ≥30 Gy bRT.** Kaplan-Meier curves illustrating the OS **(D)**, PFS **(E)**, and DSS **(F)** among patients with bulky tumors (*n*=13) treated with <30 Gy (*n*=5) vs. ≥30 Gy (*n*=8) bRT.


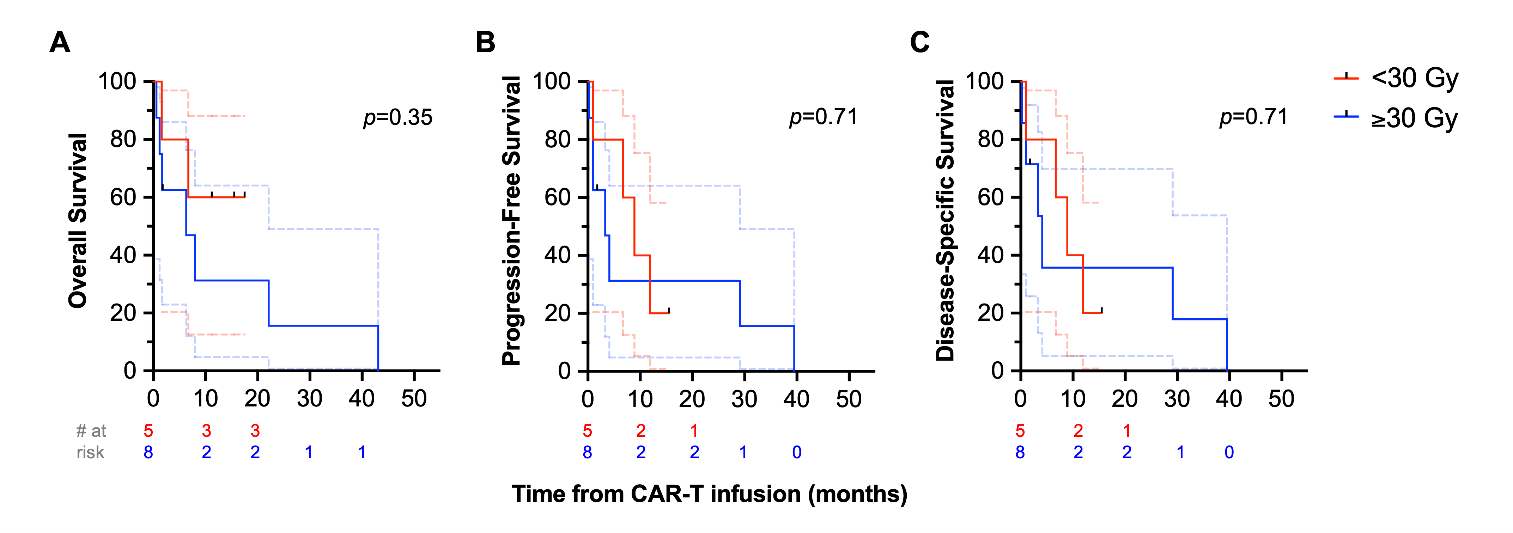

Supplement: Supplementary file 1 [file Table1.docx]
